# A human beta cell line with drug inducible excision of immortalizing transgenes

**DOI:** 10.1016/j.molmet.2015.09.008

**Published:** 2015-10-20

**Authors:** Marion Benazra, Marie-José Lecomte, Claire Colace, Andreas Müller, Cécile Machado, Severine Pechberty, Emilie Bricout-Neveu, Maud Grenier-Godard, Michele Solimena, Raphaël Scharfmann, Paul Czernichow, Philippe Ravassard

**Affiliations:** 1 Institut du cerveau et de la moelle (ICM), Biotechnology & Biotherapy Team, 75013 Paris, France; 2 CNRS UMR7225, 75013 Paris, France; 3 INSERM U1127, 75013 Paris, France; 4 Université Pierre et Marie Curie, 75013 Paris, France; 5 Endocells, Pépinière d'entreprises Institut du Cerveau et de la Moelle, 75007 Paris, France; 6 Paul Langerhans Institute of the Helmholtz Center Munich at the University Hospital and Faculty of Medicine, TU Dresden, 01307 Dresden, Germany; 7 German Center for Diabetes Research (DZD e.V), 85764 Neuherberg, Germany; 8 Max Planck Institute of Molecular Cell Biology and Genetics, 01307 Dresden, Germany; 9 INSERM, U1016, Institut Cochin, Faculté de Médecine, Université Paris Descartes, Sorbonne Paris Cité, 75014 Paris, France

**Keywords:** Cell engineering, Human pancreatic beta cell line, Conditional immortalization, Tamoxifen inducible CRE, Human beta cell function

## Abstract

**Objectives:**

Access to immortalized human pancreatic beta cell lines that are phenotypically close to genuine adult beta cells, represent a major tool to better understand human beta cell physiology and develop new therapeutics for Diabetes. Here we derived a new conditionally immortalized human beta cell line, EndoC-βH3 in which immortalizing transgene can be efficiently removed by simple addition of tamoxifen.

**Methods:**

We used lentiviral mediated gene transfer to stably integrate a tamoxifen inducible form of CRE (CRE-ERT2) into the recently developed conditionally immortalized EndoC βH2 line. The resulting EndoC-βH3 line was characterized before and after tamoxifen treatment for cell proliferation, insulin content and insulin secretion.

**Results:**

We showed that EndoC-βH3 expressing CRE-ERT2 can be massively amplified in culture. We established an optimized tamoxifen treatment to efficiently excise the immortalizing transgenes resulting in proliferation arrest. In addition, insulin expression raised by 12 fold and insulin content increased by 23 fold reaching 2 μg of insulin per million cells. Such massive increase was accompanied by enhanced insulin secretion upon glucose stimulation. We further observed that tamoxifen treated cells maintained a stable function for 5 weeks in culture.

**Conclusions:**

EndoC βH3 cell line represents a powerful tool that allows, using a simple and efficient procedure, the massive production of functional non-proliferative human beta cells. Such cells are close to genuine human beta cells and maintain a stable phenotype for 5 weeks in culture.

## Highlights

• EndoC-βH3: a conditionally immortalized human pancreatic beta cell line.
• Proliferation arrest upon removal of immortalizing transgenes with Tamoxifen.
• Enhancement of beta cell function upon removal of immortalizing transgenes.
• Tamoxifen-treated EndoC-βH3 maintain a stable phenotype for 5 weeks in culture.
• EndoC-βH3: a unique tool for large-scale drug discovery and proliferation studies.

## 1 Introduction

Type 1 diabetes results from the destruction of insulin-producing pancreatic beta cells by a beta cell-specific autoimmune process while type 2 diabetes results from the combination of insulin resistance and inadequate insulin secretion. Thus, for both types of diabetes, functional beta cell mass is not sufficient for appropriate glycemic control. Therefore understanding beta cell physiology and function is a critical issue for understanding diabetes and developing innovative therapeutic solutions. Rodent beta cells have been used so far and were instrumental for acquiring important basic knowledge of beta cell function. However, data generated with such cells cannot easily be translated to humans since major species differences have been reported [1], [2]. Thus, access to human beta cells is crucial to progress in understanding human specific beta cell function and, unfortunately, scarcity of organ donors makes it necessary to search for other sources [3].

To develop such alternative sources, large efforts have been undertaken to differentiate human embryonic or induced pluripotent stem cells (hESCs/iPSCs) towards pancreatic mature endocrine cells. Since the important original contribution of the Viacyte group to generate endocrine cells from hESCs [4], [5], recent advances have been made in this field to obtain *in vitro* more fully mature pancreatic endocrine cells [6], [7]. Still, both the production yield and the robustness of the process need to be further improved. Using an approach based on targeted oncogenesis in human fetal pancreas, we generated the first immortalized human beta cell line, referred as EndoC-βH1, giving access to unlimited number of functional human beta cells [8]. Although, this line is similar to primary adult human beta cells, it is continuously proliferating, which represents a major difference with mature beta cells that that have a low proliferation rate [9]. We recently reported the production of the second generation of human beta cell line, referred to as EndoC-βH2 that was conditionally immortalized. In this cell line, both large T antigen of SV40 (SV40LT) and human telomerase reverse transcriptase (hTERT), used as immortalizing transgenes, can be removed by CRE mediated excision [10]. We have shown that constitutive expression of CRE in EndoC-βH2 cells resulted in drastic proliferation arrest and enhancement of beta cell function both at the level of insulin content and secretion upon glucose stimulation. Thus, excised EndoC-βH2 cells are highly representative of human primary beta cells.

In previous studies [10], we transduced EndoC-βH2 cells with a lentiviral vector expressing CRE that efficiently excised immortalizing transgenes in more than 95% of cells. Although such an approach is efficient, mass production of excised cells that would require massive amounts of viral vectors cannot be easily achieved. Therefore, to circumvent this limitation, we devised a drug-activated excision strategy coupled with antibiotic selection.

Many drug-inducible systems have been used to control gene expression both *in vitro* and *in vivo* [11], [12], [13], [14]. We selected here the one based on CRE-ERT2 fusion protein [15]. CRE-ERT2 has high affinity for the 4-hydroxytamoxifen (TAM) but not for the endogenous estradiol. Therefore, the recombinase activity of CRE-ERT2 is dependent on the addition of this compound to the culture medium. After its exposure to the specific inducer TAM, CRE-ERT2 is translocated from the cytoplasm into the nucleus and excises loxP-flanked DNA regions.

In the present study, we stably modified EndoC-βH2 excisable line by lentiviral vector-mediated gene transfer to integrate both CRE-ERT2 and a constitutive puromycin selection marker. The resulting line, EndoC-βH3, was selected and massively expanded *in vitro* in the presence of puromycin. TAM dose and duration of treatment were optimized to achieve efficient immortalizing transgene excision. TAM mediated excision resulted in a sharp decrease of EndoC-βH3 cell proliferation and pronounced enhancement of beta-cell specific features such as insulin expression, storage in secretory granules and glucose stimulated secretion. These properties were maintained in culture for several weeks. Importantly, by opposition to the previous EndoC-β2 cells, the massive production of this cell line in its excised state is simple, giving access to large-scale drug discovery, proliferation studies and development of preclinical models.

## 2 Materials and methods

### 2.1 Lentiviral vectors and cell transduction

A tamoxifen inducible form of CRE (CRE-ERT2) was cloned downstream of the CMV promoter in a lentiviral backbone. Briefly, LR clonase II recombination was performed using pTrip CMV rfa-Gateway Delta U3 destination [16] vector and pENTR/D/TOPO–Cre-ERT2 entry clone. The Cre-ERT2 fragment was amplified by PCR from a plasmid kindly provided by Guilan Vodjdani (INSERM UMR1141) using the forward primer *5′CACCGGTACCCTCGAGATCGAT3′* and reverse primer *5′TCAAGCTGTGGCAGGGAAACC3′*, and the resulting PCR product was cloned into the pENTR/D/TOPO plasmid to generate the Cre-ERT2 entry clone.

The pTrip PGK puro polyA/CMV CRE-ERT2 Delta U3 was generated using pTrip CMV CRE-ERT2 Delta U3 as backbone in which a PGK puromycin resistance polyA was inserted in the reverse orientation on the 5′ side of the triplex sequence. Briefly, a linker containing EcoRI-compatible SacII, SalI BamHI MluI and EcoRI restriction sites was first inserted in the EcoRI site of pTrip CMV CRE-ERT2 Delta U3. Next, the polyA signal from human beta globin was amplified by PCR from pCDNA3.0 vector (Invitrogen) with primers containing SacII and SalI overhanging ends, and the resulting PCR product was cloned in the integrated linker sequence between SacII and SalI sites. The PGK promoter sequence was digested from pTrip PGK eGFP Delta U3 vector [17] using MluI and BamHI and cloned in the polyA containing vector. Finally, the puromycin resistance gene was amplified by PCR from pLKO puro vector (Addgene) with primers containing BamHI and SalI overhanging ends and the resulting PCR product was cloned between the PGK promoter and the polyA sequences in the corresponding restriction sites. Lentiviral vector stocks were produced by transient transfection of 293T cells with the p8.91 encapsidation plasmid, pHCMV-G, encoding the vesicular stomatitis virus (VSV) glycoprotein-G and the pTRIP ΔU3 as previously described [18].

EndoC-βH2 cells were transduced with pTrip PGK puro polyA/CMV CRE-ERT2 Delta U3 to generate EndoC-βH3 cells using a total amount of viral particles of 30 ng of p24 capside protein per 10^5^ cells in the presence of 10 μg/ml DEAE-dextran as described elsewhere [18].

### 2.2 Cell line culture and excision process

EndoC-βH3 cells were cultured in DMEM containing 5.6 mM glucose, 2% BSA fraction V, 50 μM 2-mercaptoethanol, 10 mM nicotinamide, 5.5 μg/ml transferrin, 6.7 ng/ml sodium selenite, Penicillin (100 units/ml)/Streptomycin (100 μg/ml). Ten μg/ml of puromycin (selective antibiotic) were added extemporaneously in the complete medium. Cells were seeded onto matrigel- and fibronectin-coated culture plates at 4 × 10^6^ cells/plate. Passage was performed every week. Inducible excision of CRE mediated immortalizing transgenes was performed with addition of TAM, 1 μM unless specify in the text.

Cells were counted according to manufacturer instructions using the ADAM-MC automatic cell counter instrument (NanoEnTek Inc. Seoul Korea).

### 2.3 Immunostaining

For immunocytochemistry, EndoC-βH3 cells were treated with TAM for 14 days. They were next seeded on 12-mm matrigel/fibronectin glass coated coverslips and further cultured with TAM for 7 days. Next, the cells were fixed for 1 h in 4% paraformaldehyde. The following antibodies were used for immunostaining: guinea pig anti-insulin antibody (1/500, DakoCytomation, A0564) and mouse anti-SV40LT (1/50, Calbiochem Merck Biosciences, DP-02). The secondary antibodies were fluorescein anti-mouse antibody (1/200, Immunotech, IM0819) and Texas-red anti-guinea pig antibody (1/200; Jackson Immunoresearch Laboratories, 706-076-148). Nuclei were stained with Hoechst 33342 fluorescent stain (Life Technologies). Digital images of cells were captured using an Olympus Fluoview FV1000 confocal microscope.

### 2.4 Cell proliferation assays

Cell proliferation analysis was conducted using Click-iT EdU Alexa Fluor 647 Flow Cytometry Assay Kit (Life Technology). Briefly, EdU was added into cell-culture medium to a final concentration of 10 μmol/l one hour before the endpoint of the experiments. Cells were collected after trypsin treatment, washed once with 2 ml of 1% BSA in PBS, fixed using Click-iT fixative, and incubated for 15 min in saponin-based permeabilization solution. Cells were then treated with Click-iT reaction cocktail, according to manufacturer's instruction, for 30 min before flow cytometry analysis. DAPI (1 μM) (FxCycle Violet Stain; Life Technologies) was added directly before FACS analysis. Data were acquired on an LSRFortessa (BD Biosciences) and analyzed with FACS Diva software (BD). A total of 50,000 events were collected for the cell proliferation analysis.

### 2.5 RNA isolation, reverse transcription and RT-PCR

Total RNA was isolated from EndoC-βH3 cell line using the RNeasy microkit (Qiagen), as described previously [10]. First strand cDNA was prepared using Superscript reagents (Invitrogen). Quantitative RT-PCR was performed using LightCycler 1536 DNA Green Master mix (Roche) and analyzed on a 1536 LightCycler system (Roche), according to the manufacturer's instructions. The list of primers used is presented in supporting Table 1. Qualitative RT-PCR for CRE expression analysis was performed using CRE-F AAAATTTGCCTGCATTACCG and CRE-R ATGTTTAGCTGGCCCAAATG primer pairs generating a 262 nucleotides amplification product.

### 2.6 Insulin secretion and content

EndoC-βH3 cells were seeded onto matrigel- and fibronectin-coated 96 well plates at 7 × 10^4^ cells/well for un-excised and excised cells. Three days later, cells were incubated overnight in culture medium that contained 2.8 mM glucose, followed by 60 min incubation in HEPES-buffered Krebs–Ringer Buffer (KRB) (115 mmol/l NaCl, 5 mmol/l KCl, 1 mmol/l CaCl_2_, 1 mmol/l MgCl_2_, 24 mmol/l NaHCO_3_, 10 mmol/l HEPES pH 7.4, and 0.2% BSA) that contained 0.5 mM glucose. At the end of this incubation, stimulated insulin secretion was measured by static incubation in KRB that contained varying glucose concentrations for 60 min. Glucose stimulation was performed in the presence or absence of 500 μM IBMX.

For insulin content measurement, cells were lysed directly in the culture wells with TETG solution 20 mM Tris pH 8.0; 0.1% Triton X-100; 1% Glycerol; 137 mM NaCl; 2 mM EGTA and anti-protease tablet (Roche) for 5 min on ice. The lysate was next centrifuged at 3,000 rpm for 5 min and stored at −20 °C until insulin ELISA assay.

Insulin secretion and intracellular content were measured in duplicate by ELISA according to manufacturer's instructions using the human insulin kit (Mercodia), which does not cross-react with proinsulin.

### 2.7 Electron microscopy

Cells were fixed with 2.5% Glutaraldehyde and 4% Paraformaldehyde in Sörensen's phosphate buffer (pH 7.4) over night. After fixation, cells were processed for standard Epon embedding as previously described [19]. Ultrathin sections with a thickness of 70 nm were cut on a Leica EM6 ultramicrotome and imaged using a Tecnai 12 Biotwin Transmission Electron Microscope (FEI Company, Hillsboro, OR, USA) with a bottom-mount 2 Å∼2K F214 CCD camera (TVIPS, Gauting, Germany). Sixty random images with a size of 8.92 μm × 8.92 μm were taken per condition at a resolution of 4.35 nm/pixel. TEM images were viewed using FIJI software and insulin secretory granules (SGs) were scored manually on a Wacom Cintiq 15X LCD tablet (Wacom) to calculate their number normalized for β-cell cytosolic area.

### 2.8 Oligonucleotides-based array comparative genomic hybridization (CGH array)

CGH array was performed using SurePrint G3 Human CGH Bundle (4 × 180K) (Agilent) according to manufacturer instructions. Briefly, 1 μg of genomic DNA corresponding to either a human male control [20], EndoC-βH2 or EndoC-βH3 cells both at passage 40 was fragmented by heating at 95 °C for 30 min. Fragmented DNAs were labeled with Cy3 (control DNA) and Cy5 (EndoC-βH2/EndoC-βH3 DNA) fluorescent dUTP, respectively, using Genomic DNA Enzymatic Labeling Kit (Agilent Technology). Microcon YM 30 spin columns (Millipore) were used to remove the unincorporated nucleotides and dyes. Hybridizations of labeled DNA to SurePrint G3 Human CGH Bundle (4 × 180K) array (Agilent) were performed in a hybridization oven at 42 C at 20 rpm for 40 h. Hybridized arrays were then washed following the manufacturer's instructions. Microarray slides were scanned on a Nimblegen MS200 Microarray Scanner at a 2 μm resolution. Feature extraction was done with Cytogenomics Software (Agilent). Extracted data were imported and analyzed using Nexus 7.0 (Biodiscovery).

## 3 Results

### 3.1 Production of EndoC-βH3, an excisable human beta cell line that integrated CRE-ERT2

**Figure 1:**
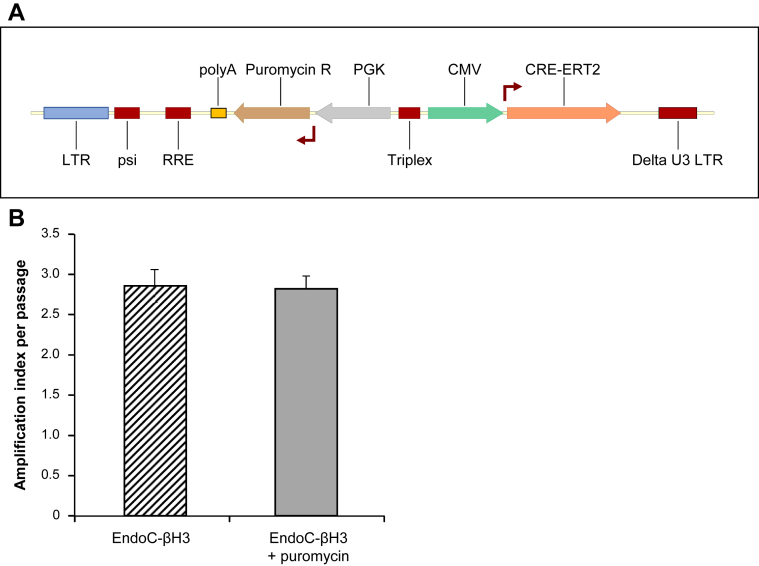
**Production of EndoC-βH3 cells a Tamoxifen inducible excisable human beta cell line derived from EndoC-βH2**. (**A**) Schematic representation of lentiviral vector used to produce EndoC-βH3 cells expressing both the TAM inducible form of CRE (CRE-ERT2) and the resistance to puromycin (**B**) Cells were passaged every week. Amplification index was defined as fold change between the initial number of seeded cells and the one counted one week later before cell passage. Data represent average of amplification index ± S.E.M. over 10 passages of EndoC-βH3 cells cultured with or without antibiotic (puromycin) selection.

Our objective was to stably integrate the coding sequence of CRE-ERT2 fusion protein into the genome of EndoC-βH2 cells and to couple such integration with an antibiotic resistance to select CRE-ERT2 positive cells. First, EndoC-βH2 sensitivity to neomycin, puromycin or hygromycin antibiotics was tested. Both puromycin and hygromycin efficiently killed EndoC-βH2 cells within two weeks at 10 μg/ml and 150 μg/ml respectively (not shown). We next constructed a lentiviral vector expressing both CRE-ERT2 and a Puromycin resistance gene under the control of CMV and PGK promoters, respectively (Figure 1A). The vector was used to transduce EndoC-βH2 cells, and we named the resulting puromycin-selected cells EndoC-βH3. Importantly, the proliferation rate of EndoC-βH3 cells was similar in the presence or absence of antibiotic (Figure 1B), indicating that the selection does not hamper the overall growth of the cells. EndoC-βH3 can therefore be massively amplified in culture. We next analyzed the chromosomal stability of EndoC-βH3 cells at passage 40 in comparison to EndoC-βH2 cells. CGH array profiles are almost identical between the two lines (Figure S1) indicating chromosomal stability following integration of CRE-ERT2 and antibiotic selection.

### 3.2 Tamoxifen treatment to achieve efficient activation of recombinase CRE and excision of SV40LT

**Figure 2:**
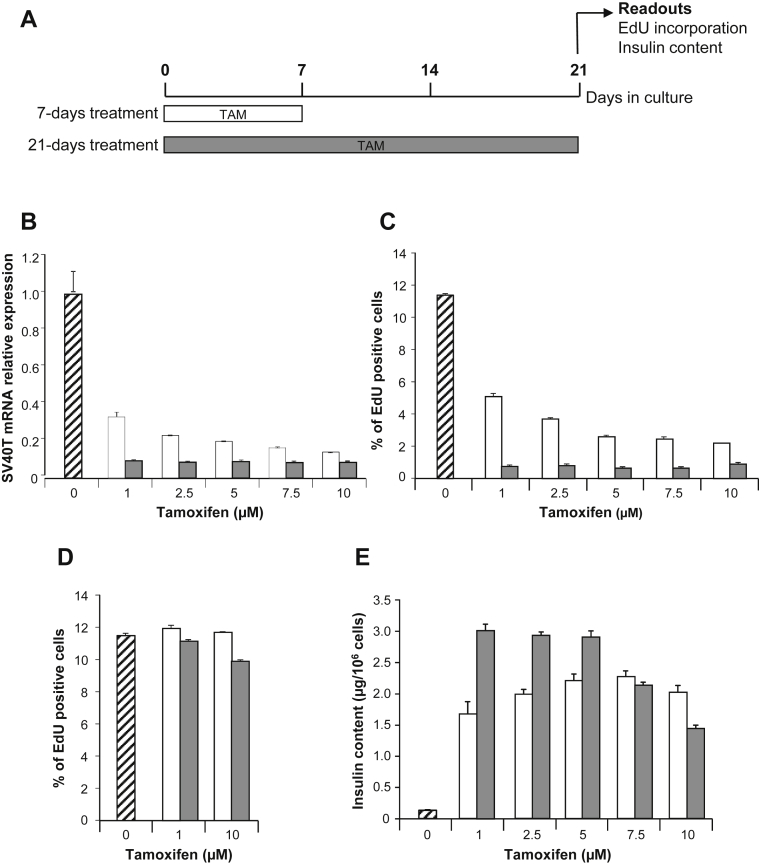
**Definition of optimal procedure for efficient Tamoxifen treatment of EndoC-βH3**. (**A**) Schematic representation of the experimental optimization procedure for TAM treatment. (**B**, C, E) EndoC-βH3 cells were treated for 7 days (white box) or 21 days (Gray box) with TAM. Un-treated cells are represented as hatched box. Proliferation assays and insulin content measurements were performed at day 21. (**B**) Effect of TAM treatment on SV40LT gene expression. Quantitative PCR results are representative of 2 independent experiments performed in triplicates. Data corresponded to average relative expression values normalized to TPB expression ± S.E.M. (**C**–**D**) Effect of TAM treatment on EdU incorporation by EndoC-βH3 cells (**C**) or EndoC-βH2 (**D**) relative to total cell number. EdU was added for 1 h and analysis was performed by FACS. Results are representative of 2 independent experiments performed in duplicates. Data are expressed as average percentage ± S.E.M. (**E**) Effect of TAM treatment on insulin content in EndoC-βH3. Data are expressed as insulin content per million cells ± S.E.M. of 3 independent wells per condition assayed in triplicates by ELISA.

Our previous data obtained with EndoC-βH2 indicate that constitutive CRE expression results in excision of SV40LT [10]. Under such conditions, 21 days after transduction with CRE-expressing lentiviral vector, proliferation was massively reduced and insulin content increased. To follow excision efficacy in EndoC-βH3 cells that express CRE-ERT2, we used 3 readouts: SV40LT expression, incorporation of EdU for proliferation assessment and insulin content measurement. Both the concentration of TAM and the duration of treatment were investigated. Excision efficacy was monitored 21 days after TAM addition using increasing doses from 1 to 10 μM. TAM was maintained in the medium for 7 or 21 days (Figure 2A). Importantly, the decrease in SV40LT expression and EdU incorporation were parallel (Figure 2B–C). With 7 days treatment, EndoC-βH3 proliferation decreases in a dose-dependent manner. 11.5% of control untreated cells incorporated EdU whereas EdU incorporation decreased to 5.1% and 2.2% with 1 and 10 μM TAM, respectively. With 21 days TAM treatment, all tested concentrations were equivalent and, on average, only 0.75% of cells incorporated EdU (Figure 2C). EndoC-βH2 cells that do not contain an integrated TAM inducible CRE were treated for 7 or 21 days with either 1 or 10 μM TAM. Such treatments had no effect on cell proliferation (Figure 2D) demonstrating that the massive reduction of proliferation observed with EndoC-βH3 treated with TAM is due to the induction of CRE-ERT2 dependent excision.

**Figure 3:**
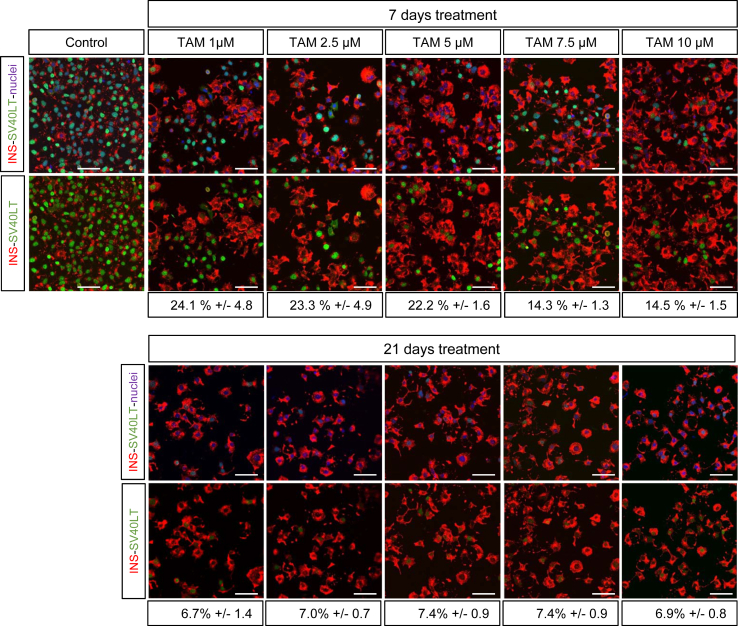
**SV40LT and insulin immunostaining following Tamoxifen treatment**. SV40LT (green), Insulin (red) Nuclei (blue) immunofluorescent staining of control or excised cells analyzed 21 days after a 7 or 21 days TAM treatment. Percent of SV40LT positive nuclei relative to total number of nuclei is indicated below each conditions. Confocal acquisition settings were identical for all conditions. Scale bar = 50 μm.

Following a 7 days TAM treatment, insulin content increased by 12.9 and 17.5 fold when treated with 1–10 μM TAM compared to control untreated cells (Figure 2E). Such induction was lower than the 23.3 fold induction obtained with 21 days 1 μM TAM treatment (Figure 2E). Similar high inductions were obtained with 2.5 and 5 μM TAM with 21 days TAM treatment protocol whereas higher doses were less efficient (Figure 2E). Therefore 1 μM TAM treatment for 21 days represented the optimal dose that gave the highest outcome to decrease SV40LT expression, to decrease proliferation and to enhance insulin content. Finally, the percentage of cells positive for SV40LT protein expression sharply decreased upon 21 days TAM treatment. Indeed with the optimal TAM treatment, SV40LT gene expression represents only 8.6% of untreated EndoC-βH3 cells (Figure 2B) and the percentage of SV40LT expressing cells is reduced to 6.7% (Figure 3).

### 3.3 EndoC-βH3 function after Tamoxifen-mediated transgene excision

**Figure 4:**
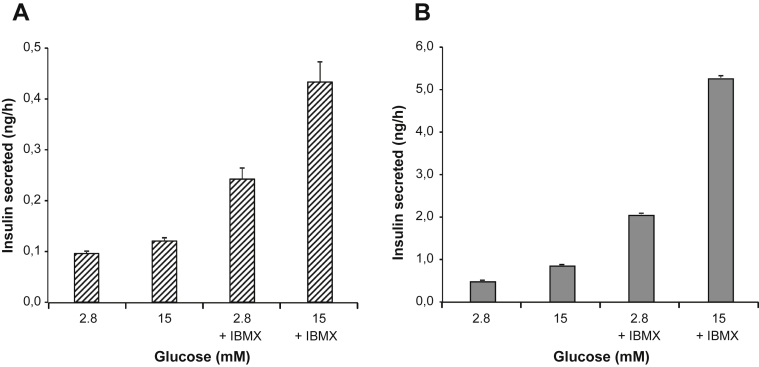
**Glucose responsive insulin secretion in EndoC-βH3 following treatment with Tamoxifen**. EndoC-βH3 were treated or not for 21 days with 1 μM TAM. (**A**) Glucose stimulated insulin secretion (GSIS) on un-treated EndoC-βH3 in presence or absence of IBMX. (**B**) GSIS on TAM-treated EndoC-βH3 in presence or absence of IBMX. (**A**–**B**) GSIS data are expressed as ng of secreted insulin per hour ± S.E.M. of 3 independent wells seeded with 7 × 10^4^ cells per condition assayed in triplicates by ELISA.

**Figure 5:**
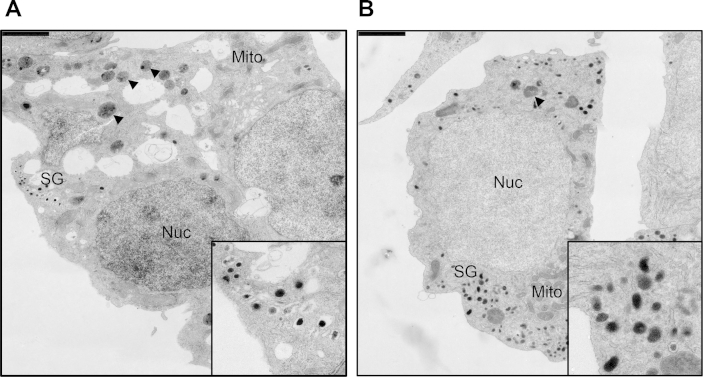
**TEM analysis of treated and untreated EndoC-βH3**. Non-treated and treated EndoC-βH3 cells were analyzed after Epon embedding and ultrathin sectioning. (**A**) Non-treated EndoC-βH3, inset: detail of insulin SGs, (**B**) TAM-treated EndoC-βH3, inset: detail of insulin SGs. Scale bar: 2 μm. Labeling key: Nuc, nucleus; Mito, mitochondria; SG, secretory granules; arrowheads, degradative compartments.

Upon TAM-mediated excision, insulin content in EndoC-βH3 sharply increased when compared to non-excised cells (Figure 2D). We asked whether insulin secretion remained responsive to glucose stimulation. Static glucose-stimulated insulin secretion was assayed on exact same number of seeded EndoC-βH3 cells that had been treated or not with TAM for 21 days. As for non-excised EndoC-βH2 cells, glucose did not stimulate insulin secretion in the absence of IBMX, a phosphodiesterase inhibitor that increases intracellular levels of cAMP. In the presence of IBMX, insulin secretion was stimulated 1.8 fold (stimulation index) at 15 mM Glucose compared to 2.8 mM (Figure 4A). When excised with the optimal TAM treatment defined above, EndoC-βH3 cells responded to glucose both in absence and presence of IBMX with a stimulation index of 1.8 and 2.6 respectively (Figure 4B). Importantly, we tested the robustness of both insulin content and glucose responsive insulin secretion in the 21 days TAM treated cells at different passages spanning from passage 36 to 60. On average, from 6 independent experiments, insulin content was of 3.1 ± 0.41 μg of insulin per million cells that represents 10–20% of the content found in primary human Islets [21]. The stimulation indexes obtained in absence of IBMX were 1.8 ± 0.35 (S.E.M.) and 2.6 ± 0.15 (S.E.M) in the presence of IBMX. This indicated that insulin content and secretion in TAM treated EndoC-βH3 cells were stable over passages. TEM imaging of TAM treated and non-treated EndoC-βH3 cells corroborated the presence of a significantly higher number of insulin SGs in TAM treated cells (13.94 ± 1.56 (S.E.M) SGs/10 μm^2^) compared to non-treated cells (6.53 ± 0.83 (S.E.M.) SGs/10 μm^2^) (Student's t-test p-value: 4.77325 × 10^−5^) (Figure 5). Also, untreated cells contained more degradative compartments compared to treated cells.

### 3.4 Long-term phenotype of Tamoxifen excised EndoC-βH3 cells

**Figure 6:**
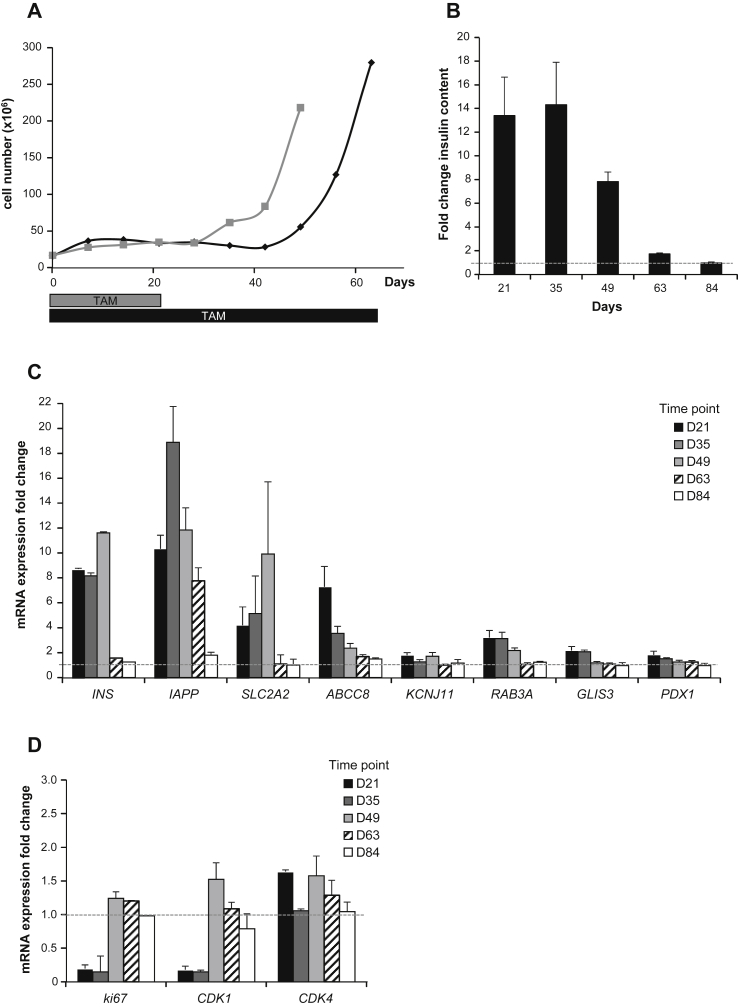
**Long-term phenotype of Tamoxifen treated EndoC-βH3**. (**A**) Cell growth monitoring over time (every 7 days) during 63 days. TAM treatment was either stopped at day 21 (in gray), or continued during the whole experiment (in black). (**B**) Insulin content was determined in EndoC-βH3 cells continuously treated with TAM and compared to untreated cells. Analysis was performed every second week, starting 21 days after initial TAM treatment. Data are expressed as fold change over untreated cells (dotted line) of insulin content ± S.E.M. of 3 independent wells per condition assayed in replicates by ELISA. (**C**–**D**) Gene expression by quantitative RT-PCR was determined in EndoC-βH3 cells that were continuously treated with TAM and compared to untreated cells. Analysis was performed every second week, starting 21 days after initial TAM treatment. Data are expressed as fold change of mRNA expression relative to TBP ± S.E.M. of 3 independent RNA preparations assayed in triplicates.

We followed TAM-treated EndoC-βH3 cells over time. Total cell number was counted every 7 days to follow cell growth (Figure 6A). When TAM was removed at day 21, cell number remained stable for one additional week (up to day 28 of culture) and then started to increase (Figure 6A, gray line). When TAM was continuously maintained in the culture medium, cell number remained stable for an additional week and thus lasted 5 weeks in total (Figure 6A, black line). We next monitored insulin content over time (Figure 6B). At days 21 and 35, cells constitutively treated with TAM contained 13.3 and 14.3 times more insulin that the non-treated cells, respectively. The increase in insulin content was therefore almost constant during the whole period. However, it decreased to 7.8 fold by day 49, suggesting that with time, proliferating non-excised cell that escaped excision, and contained low levels of insulin, expand. This effect was further enhanced at day 84 (Figure 6B)

We monitored gene expression in TAM-treated EndoC-βH3 cells over time. Insulin mRNA expression was consistent with insulin content measurements (Figure 6C). Indeed, insulin mRNA levels in TAM-treated cells were at least 8 fold higher until day 49 when compared to non-excised cells (Figure 6C). By day 63 insulin expression levels in TAM-treated cells returned to the one found in untreated cells. We also assessed in TAM-treated EndoC-βH3 cells the expression of a number of genes found to be significantly increased by constitutive CRE excision of EndoC-βH2 cells [10]. Their expression was in line with the above-described observations. Expression levels of beta cells enriched genes such as *IAPP*, *SLC2A2*, *KCNJ11*, *ABCC8*, *RAB3A*, *GLIS3* and *PDX1* remained higher in TAM exposed cells compared to un-treated cells until day 49 and returned to the un-excised level by day 63 (Figure 6C). Moreover, expression of genes involved in cell cycle control such as *Ki67* and *CDK1* was massively reduced to 0.17 and 0.16 fold, respectively, in TAM treated cells when compared to un-treated cells. This reduction was seen only until day 35 (Figure 6D). As observed in EndoC-βH2 upon CRE excision, CDK4 was also not modulated with TAM treatment (Figure 6D).

## 4 Discussion

Collectively, after 21 days TAM treatment, the phenotype of EndoC-βH3 cells is not significantly different from the one previously reported for EndoC-βH2 expressing constitutive CRE for 21 days [10] (Supplemental Table S2). This observation is true when monitoring: (i) cell growth; (ii) SV40LT deletion; (iii) insulin content and insulin secretion upon glucose stimulation; (iv) expression of important functional beta cell and proliferation markers. Thus, by developing this simple and efficient TAM treatment procedure, non-proliferative functional human beta cells can now be produced in large quantity. It has been reported that TAM could have proapoptotic effect *in vivo* on mouse beta cells [22]. We addressed cell survival upon continuous exposure to TAM (1 μM and 10 μM) for 21 days in EndoC-βH2, the cell line from which EndoC-βH3 was derived, and in which the CRE-ERT2 transgene is absent. We did not observe any difference in cell growth between treated and non-treated cells (Supplemental Figure S2) nor at the level of EdU incorporation (Figure 2C) even at the high dose of 10 μM. Our results thus indicate that continuous treatment with TAM does not interfere with cell survival. Morphometry of TEM images revealed a 2.15 fold increase in the number of insulin SGs in TAM treated vs. non-treated cells. Treated cells contained instead fewer degradative compartments compared to non-treated cells, possibly due to the switch from proliferation to SG production.

In previous work, when CRE was constitutively expressed in EndoC-βH2 cells, immortalizing transgenes were excised in the vast majority of cells [10]. We hypothesized that the remaining non-excised cells had escaped initial transduction with CRE-expressing lentiviral vector. Therefore, it was reasonable to envisage that adding an antibiotic selection system in the integrative vector expressing CRE-ERT2 would allow selection of 100% CRE-ERT2 positive cells and increase efficiency of immortalizing transgene excision. When dose and duration of TAM treatment were optimized, we observed that, although cells were grown under continuous puromycin selection pressure, efficacy of excision was not complete since some cells remained in a proliferative state (Figure 2A) and scattered cells still expressed SV40LT (Figure 3). Two main reasons could explain this counterintuitive observation of incomplete CRE mediated recombination. First, TAM-inducible CRE recombination has been extensively used *in vivo* in transgenic models and efficacy of recombination is highly variable depending on recombinase expression levels [23], [24] and genome availability of LoxP sites [25], [26], [27]. Since, EndoC-βH3 cells were generated from lentiviral mediated transduction of EndoC-βH2 cells without further clonal selection, the variability of CRE-ERT2 integration sites may be responsible for inter-cellular variability of recombinase expression. Such heterogeneity could sustain the fact that a small cell population was unable to achieve efficient excision of immortalizing transgenes. Second, the lentiviral construct used to generate EndoC-βH3 cells is composed of two separate transcription units. Specifically, in the same integrated fragment, CRE-ERT2 is expressed under the control of the CMV promoter and the resistance to puromycin under a different promoter (PGK). Thus, with time in culture, CRE-ERT2 transcription could be reduced or extinguished, while puromycin resistance remains selected by the antibiotic in the culture medium. Of note, CMV promoters are known to be subject to extinction with time [17], [28], [29], [30].

While the vast majority of the cells efficiently recombine the loxP sites and excise the immortalizing transgenes, rare cells escape this process. Our data indicate that the overall composition of the cell populations (unexcised vs excised cells) is maintained relatively constant for 49 days with the majority of cells baring a non-proliferative state with high insulin content. However, with time, the proliferative advantage of non-excised cells will result in a time dependent modification of the relative proportion of both cell populations and by day 66, the cell population is mainly composed of cells that behave like proliferating, untreated EndoC-βH3. Interestingly, although such cells were proliferating in the presence of TAM, after 66 and 119 days of TAM treatment, they still expressed CRE-ERT2 mRNA (Figure S3), suggesting that they were unable to activate loxP recombination to efficiently remove immortalizing transgenes. The above-described experiments were performed on non-clonal cell populations. Amplifying cell subclones could represent a way to select lines with further increased loxP recombination efficacy. Furthermore, complete removal of proliferating SV40LT positive cells could be achieved through counter-selection using a suicide gene approach such as thymidine kinase in the presence of Gancyclovir [31].

## 5 Conclusion

Altogether, we have produced a novel human beta cell line derived from EndoC-βH2 cells [10] that contains floxed immortalizing transgenes and an integrated TAM inducible form of CRE recombinase. Such line can be massively amplified followed by immortalizing transgenes removal by simple addition of TAM, giving rise to non-proliferating functional human beta cells. This new line offers simple and efficient procedure to mass produce non-proliferative human beta cells that can maintain a stable phenotype for 5 weeks in culture thus providing a powerful tool for large-scale drug discovery.
