# Supplementary material for: A human beta cell line with drug inducible excision of immortalizing transgenes
Source: Mol Metab. 2015 Oct 20;4(12):916–25. doi: 10.1016/j.molmet.2015.09.008 (PMC4731729; doi:10.1016/j.molmet.2015.09.008)
Supplement: Supplementary file 1 [file mmc1.docx]

**SUpplementary material**

**Supplemental Table S1: list and sequences of primers used for Quantitative RT PCR**

| **Gene** | **Forward primer (5’ to 3’)** | **Reverse primer (5’ to 3’)** |
| --- | --- | --- |
| *TBP* | TGCACAGGAGCCAAGAGTGAA | CACATCACAGCTCCCCACCA |
| *Insulin (INS)* | TCCAGGACAGGCTGCATCA | CCATGGCAGAAGGACAGTGA |
| *IAPP* | ACATGTGGCAGTGTTGCATT | TCATTGTGCTCTCTGTTGCAT |
| *SLC2A2* | ATCCAAACTGGAAGGAACCC | CATGTGCCACACTCACACAA |
| *ABCC8* | GCCCACGAAAGTTATGAGGA | AAGGAGATGACCAGCCTCAG |
| *KCNJ11* | GCTTGCTGAAGATGAGGGTC | CTCATCGTGCAGAACATCGT |
| *RAB3A* | CATAGCGCCCCGGTAGTAT | GACCATCTATCGCAACGACA |
| *GLIS3* | ACAACCCCTCCTCCCAGTTA | TGATGTGGTGAGGAGATGGA |
| *PDX1* | TACTGGATTGGCGTTGTTTGTGGC | AGGGAGCCTTCCAATGTGTATGGT |
| *Ki67* | AAGGAACAGCCTCAACCATCAGGA | CCAAGCTTTGTGCCTTCACTTCCA |
| *CDK1* | CAGAGCTTTGGGCACTCCCAATAA | GCTAGGCTTCCTGGTTTCCATTTG |
| *CDK4* | TCAAGGTAACCCTGGTGTTTGAGC | GGCGCATCAGATCCTTGATCGTTT |
| *SV40LT* | TGCCTGGAACGCAGTGAGTTTT | AACTCAGCCACAGGTCTGTACCAA |

**Supplemental Table S2: side by side comparison between EndoC-βH2 and EndoC-βH3**

|  | EndoC-βH2 + LvCRE (21 days) | EndoC-βH3 + TAM (21 days) |
| --- | --- | --- |
|  |  |  |
| SV40LT expression relative to non-excised cells (%) | 8.4 +/- 0.21 | 8.6 +/- 0.24 |
|  |  |  |
| Insulin content (µg per 10^6^ cells) | 2.3 +/- 0.20 | 3.1 +/- 0.41 |
|  |  |  |
| Stimulation index in absence of IBMX | 2.1 +/- 0.26 | 1.8 +/- 0.35 |
| Stimulation index in presence of IBMX | 2.4 +/- 0.22 | 2.6 +/- 0.15 |

Stimulation index is defined as fold change of secreted insulin between 2.8 mM and 15 mM glucose concentration

**Supplemental Figure S1: CGH array profiles reveal chromosomal stability following integration of CRE-ERT2 and antibiotic selection**

CGH arrays were performed on EndoC-βH2 and EndoC-βH3 cells both at passages 40. Male human genomic DNA was used as a reference. Genomic DNA was probed on Agilent SurePrint G3 Human CGH Bundle (4x180K) arrays.

**Supplemental Figure S2: EndoC-βH2 cell growth monitoring for 21 days upon continuous treatment with TAM**

EndoC-βH2 cells were continuously treated with either 1 µM or 10µM of TAM for 21 days and cell growth was monitored every week and compared to un-treated EndoC-βH2.

**Supplemental Figure S3: Expression of Cre by RT PCR in EndoC-βH3 cells continuously treated with TAM for long time period**

Total RNA was extracted from EndoC-βH3 cells that were treated with TAM for 66 and 119 days in culture. As controls, RNA from EndoC-βH2 were used either naïve (negative control) or transduced with a lentiviral vector expressing CRE (LvCRE positive control). To rule out possible contamination with genomic DNA in the RNA preparation that could be amplified by PCR, the amplification was carried out using cDNA produced in the presence (+) or absence (-) of reverse transcriptase (RT).
