# Supplementary figures and images for: A human beta cell line with drug inducible excision of immortalizing transgenes
Source: Mol Metab. 2015 Oct 20;4(12):916–25. doi: 10.1016/j.molmet.2015.09.008 (PMC4731729; doi:10.1016/j.molmet.2015.09.008)

## Slide 1
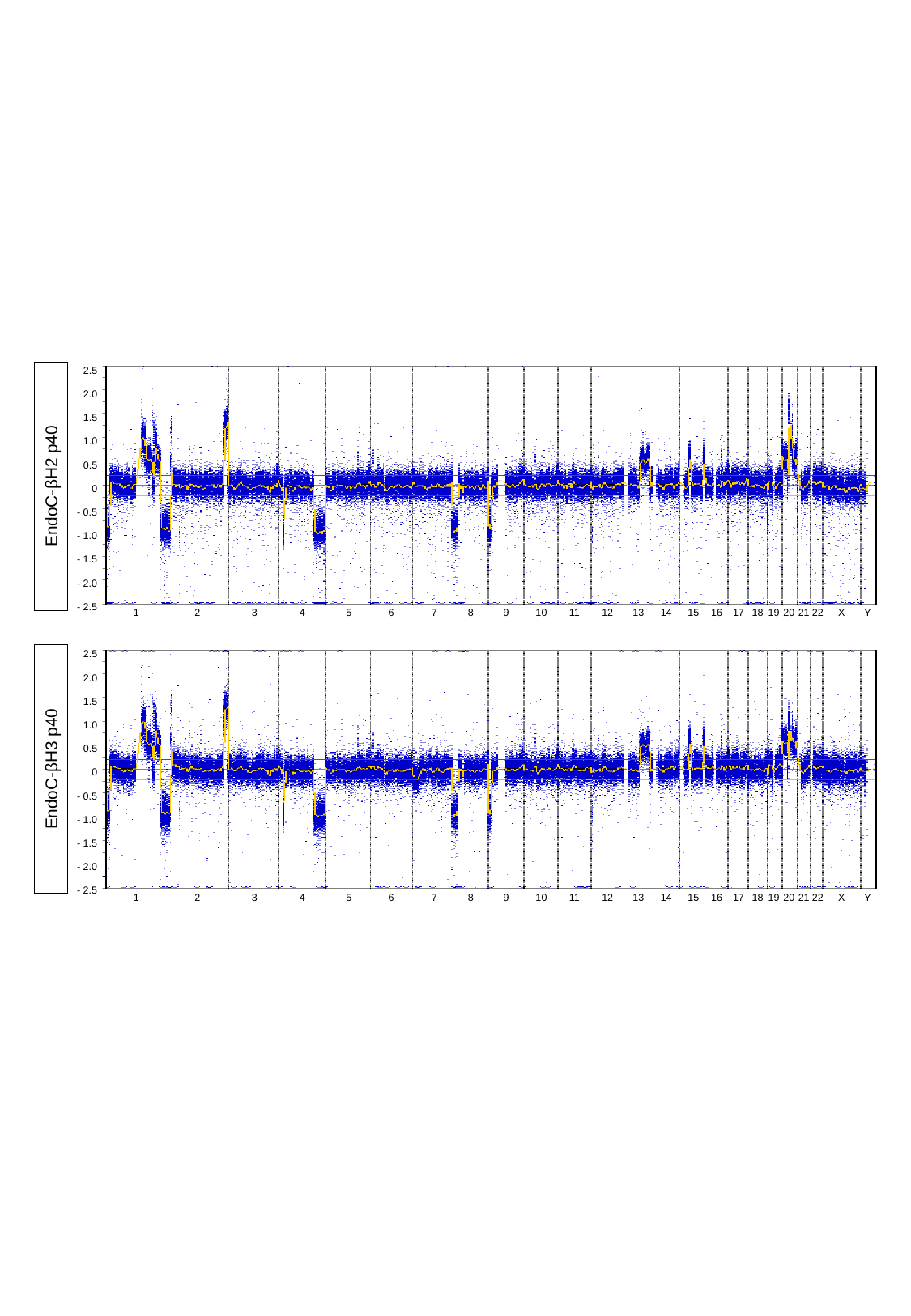

2.5
2.0
1.5
1.0
0.5
0
- 0.5
- 1.0
- 1.5
- 2.0
- 2.5
EndoC-βH2 p40
1
2
3
4
5
6
7
8
9
10
11
12
13
14
15
16
17
18
19
20
21
22
X
Y
2.5
2.0
1.5
1.0
0.5
0
- 0.5
- 1.0
- 1.5
- 2.0
- 2.5
EndoC-βH3 p40
1
2
3
4
5
6
7
8
9
10
11
12
13
14
15
16
17
18
19
20
21
22
X
Y

Supplement: Supplementary file 2 [file mmc2.pptx]

## Slide 1
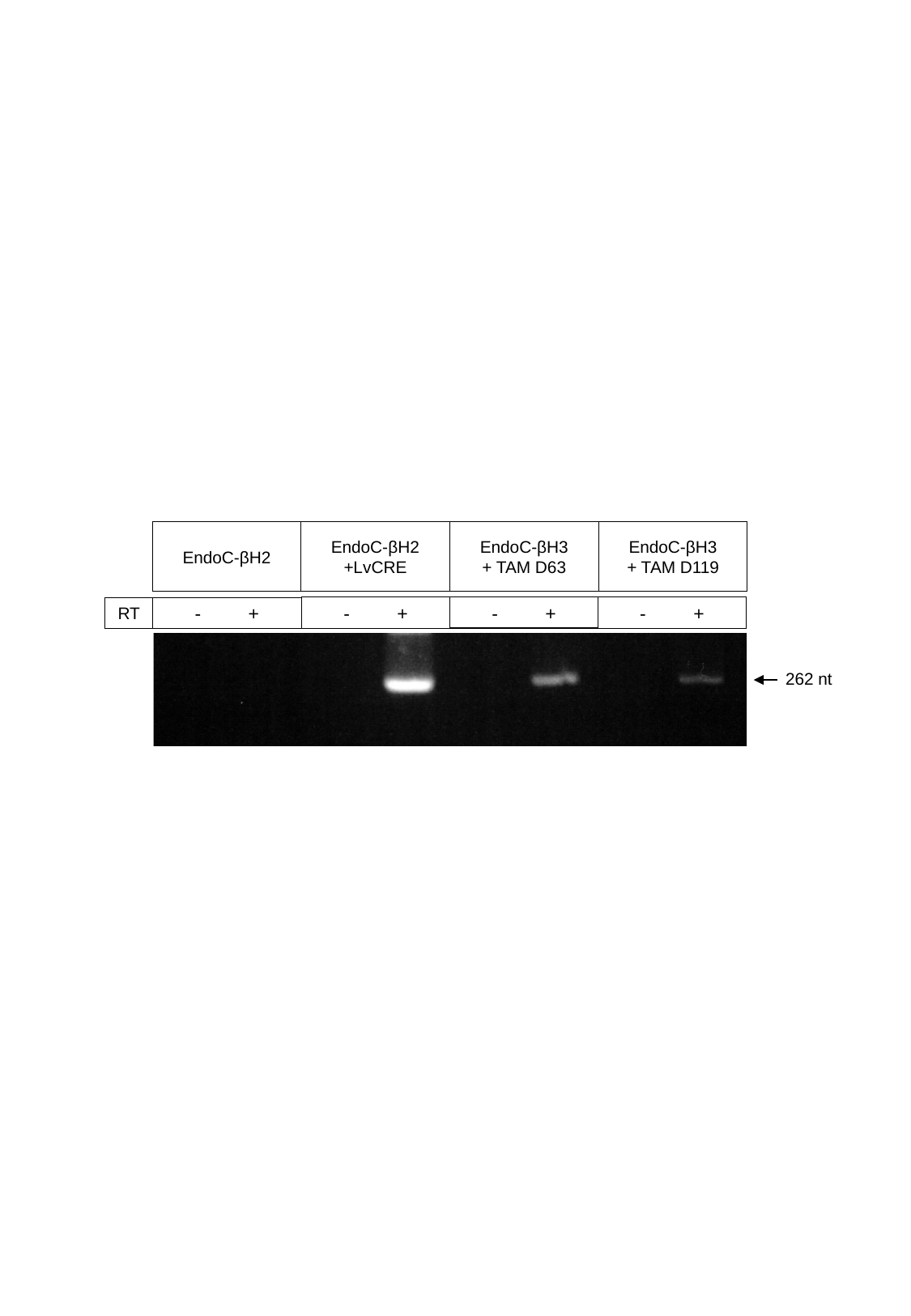

EndoC-βH2
EndoC-βH2 +LvCRE
EndoC-βH3
+ TAM D63
EndoC-βH3
+ TAM D119
- +
- +
- +
- +
RT
262 nt

Supplement: Supplementary file 4 [file mmc4.pptx]
