# Supplementary material for: A human beta cell line with drug inducible excision of immortalizing transgenes
Source: Mol Metab. 2015 Oct 20;4(12):916–25. doi: 10.1016/j.molmet.2015.09.008 (PMC4731729; doi:10.1016/j.molmet.2015.09.008)
Supplement: Supplementary file 3 [file mmc3.pptx]

## Slide 1
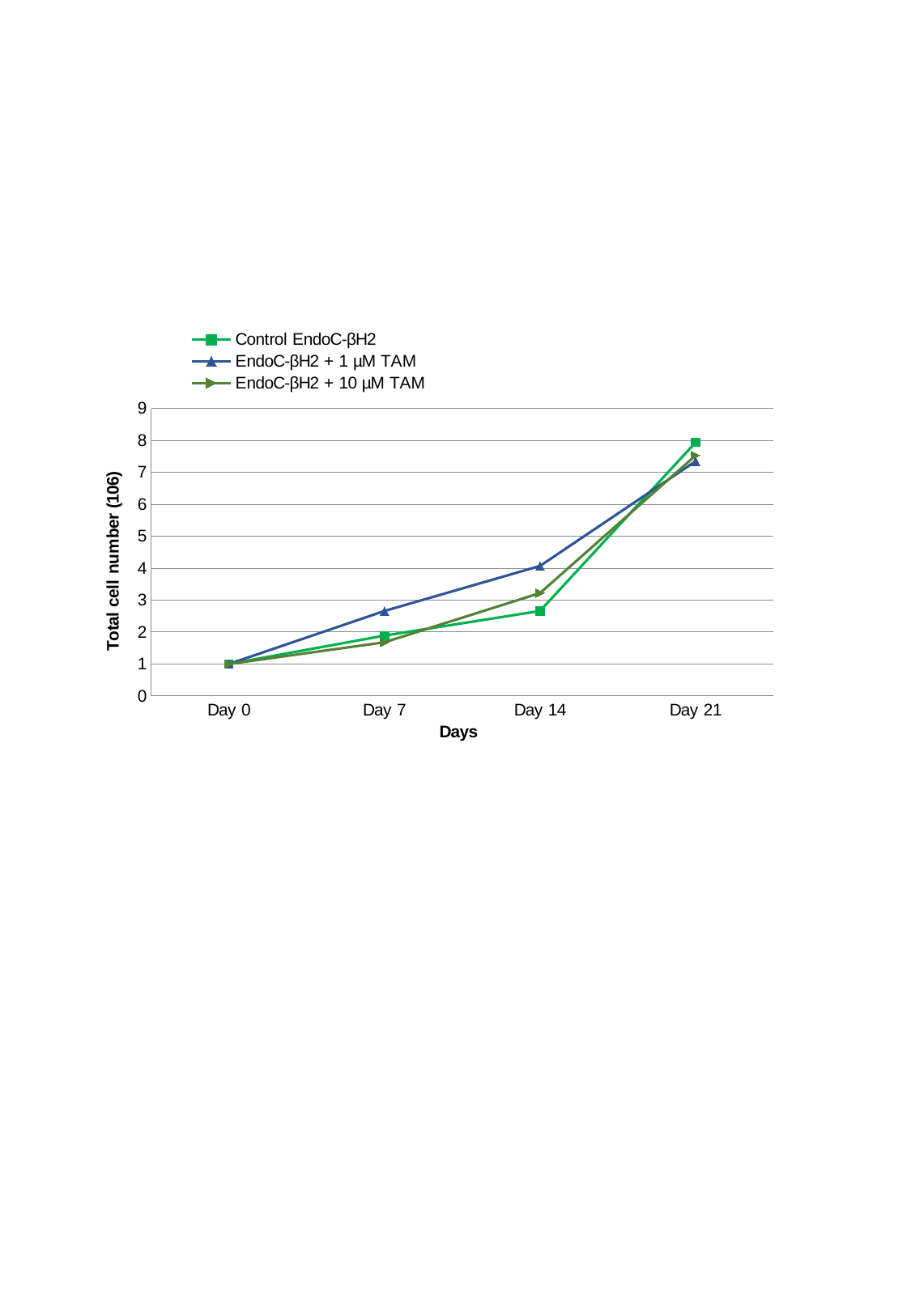

### Chart
| Category | Control EndoC-βH2 | | | EndoC-βH2 + 1 µM TAM | EndoC-βH2 + 10 µM TAM |
|---|---|---|---|---|---|
| Day 0 | 1.0 | None | None | 1.0 | 1.0 |
| Day 7 | 1.89 | None | None | 2.66 | 1.68 |
| Day 14 | 2.66 | None | None | 4.07 | 3.22 |
| Day 21 | 7.94 | None | None | 7.35 | 7.53 |
